# Addressing Global Disparities in Cervical Cancer Burden: A Narrative Review of Emerging Strategies

**DOI:** 10.1007/s11904-025-00727-2

**Published:** 2025-02-21

**Authors:** Kalpana Gopalkrishnan, Roksana Karim

**Affiliations:** Department of Population and Public Health Sciences, Keck School of Medicine, University of Southern California, Los Angeles, CA USA

**Keywords:** Cervical cancer, Screening, Low to middle income country, Technology

## Abstract

**Purpose of Review:**

Cervical cancer burden is disproportionately higher in low to middle income countries, especially in countries with a high human immunodeficiency virus (HIV) burden. This review investigates barriers to implementation and assesses current progress in cervical cancer screening in lower resource settings by reviewing technologies and strategies that have already been implemented in low to middle income countries.

**Recent Findings:**

Several novel innovations embrace the recent World Health Organization (WHO) update to screening guidelines that recommends a “screen and treat” approach rather than a “screen, triage and treat” approach. Innovations include human papillomavirus (HPV) self-sampling, portable cervical visualization devices, and creative large-scale approaches to increase screening accessibility.

**Summary:**

Overall, a low-cost, accurate, point-of-care screening test could alleviate most of the barriers associated with cervical cancer screening in lower resource settings. Further research into the development of a low-cost HPV test in conjunction with the HPV vaccine and other screening tools could expedite progress.

## Key Takeaways

1. Cervical cancer disproportionately affects women in low to middle income countries.
2. There are multiple barriers to implementing effective screening regimens at the level of the individual, community, and health care system.
3. The HPV DNA test remains the most efficacious and cost-effective method for cervical cancer screening, but cost continues to be a barrier to implementation.
4. Novel screening technologies such as self-sampling, urine testing, portable devices, and reliance on mobile and artificial intelligence technology can allow for point-of-care, affordable screening.
5. Several low-to-middle income countries have implemented novel screening guidelines that have led to higher screening rates.
6. HIV infection and prevalence remains a key consideration when developing cervical cancer screening programs in affected countries.

## Introduction

The fight against cervical cancer is one against global inequity. While cervical cancer is the fourth-most common cancer for women across the globe, there are striking global disparities in disease and mortality burden. Worldwide, in 2020, there were 13.3 cases of cervical cancer for every 100,000 women [1]. However, there was a stark disparity in cervical cancer burden between higher resource and lower resource countries, with 90% of all new cases of cervical cancer in low to middle income countries in 2020, with the highest burden in sub-Saharan Africa, followed by south and Southeast Asia, South America, and Latin America [2, 3]. To illustrate this point, Switzerland, a high income country, had the lowest mortality rate in 2020, with 1 death from cervical cancer each year for every 100,000 women, while Eswatini, a country in southern Africa, had a mortality rate of 55.7 deaths for every 100,000 women [1].

It is essential to emphasize that cervical cancer is a preventable cancer, which underscores the importance of preventive measures in reducing the global disparity of cases and deaths [4]. In 2018, the World Health Organization (WHO) Director-General announced a goal to eliminate cervical cancer by 2030, meaning that countries should have an annual incidence rate of less than 4 per 100,000 women [5]. As of 2020, only 12 out of 185 countries had age-standardized cervical cancer incidence rates that met this goal, including Switzerland and Malta [1]. Densely populated countries like India and China had higher age-standardized incidence rates of cervical cancer of 18.0 and 10.7 respectively, while countries in parts of Africa like Malawi had incidence rates as high as 67.9 [1]. With this goal in mind, the WHO announced “90-70-90” targets, aiming for every country: (1) 90% of girls have the human papillomavirus (HPV) vaccination by age 15 years, (2) 70% of women are screened for cervical cancer by a high-performing test at 35 and 45 years, and (3) 90% of women with cervical disease receive treatment [4].

However, the road to cervical cancer elimination is longer and more difficult in lower resource settings. According to the WHO, while 81% of countries released cervical cancer screening policies and plans, only 41% have an actual funded strategy in operation [5, 6]. In addition, while 125 countries had implemented an HPV vaccination program as of 2021, only 21% of girls worldwide were vaccinated for HPV as of 2022 [7, 8]. Furthermore, the COVID pandemic paused or halted implementation of many vaccination and screening programs, further slowing progress [9].

## Current Screening Protocols

Careful attention must be paid to the screening strategy in a lower resource setting, as the screening protocols that are most effective in high income countries are not the most effective in low to middle income countries. Since almost all cervical cancers are linked with HPV infection, HPV vaccination confers primary prevention of most cervical cancers. In terms of secondary prevention or screening, commonly used techniques are cytology (the Papanicolaou or “pap” smear), visual inspection with acetic acid, and HPV DNA testing. Cytology and visual inspection rely on visual examination of cervical abnormalities. Visual inspection with acetic acid examines cervical tissue with the naked eye with the assistance of acetic acid, while the pap smear examines stained cervical cells with a microscope. Visual inspection and cytology have limitations due to the variability of result interpretation between readers [10, 11]. On the other hand, HPV DNA testing identifies the causal agent of cervical abnormalities. However, HPV DNA testing is costly and not universally available.

In high-income countries, the general cervical cancer prevention practice is to “screen, triage, and treat [12].” In the United States, for example, the U.S. Preventative Services Task Force recommends that all women aged 21–29 years undergo a pap smear every three years [13, 14]. For women ages 30–65 years, the U.S. Preventative Services Task Force recommends either a pap smear every three years, an HPV DNA test every five years, or co-testing with a pap smear and an HPV DNA test every five years [14]. When a woman has an “abnormal” screening test, the American College of Obstetricians and Gynecologists recommends further triage through colposcopy, or a biopsy of cervical tissue [13]. Through colposcopy, an abnormality is further characterized to determine if or what treatment is needed, thereby reducing the risk of unnecessary treatment.

While a screen-triage-treat approach functions well in countries like the U.S., this clinical algorithm requires multiple points of contact between a patient and a provider. Moreover, in lower resource settings screening is often opportunistic, making it up to the patient to seek out screening. On the other hand, higher-income countries like the United Kingdom, Australia, and Sweden have had long-established organized screening programs with one nationwide data registry [15]. Systemic barriers and a lack of unifying screening strategies make a screen-triage-treat approach less feasible in lower resource settings.

With lower resource settings in mind, in 2021 the WHO broadly recommended an HPV DNA test with or without triage as the preferred screening strategy, meaning that a woman does not necessarily have to undergo a confirmatory colposcopy after an initial positive HPV test before treatment [16]. By endorsing a “screen and treat” strategy, WHO promoted more equitable screening protocols that reflected disparities in resource availability [17]. However, low to middle income countries are still faced with a choice of what screening and treatment methods to implement. For example, in certain countries or regions with a high HIV prevalence, women living with HIV have a higher likelihood of being identified as HPV-positive [18]. While women with HIV have a 6 times higher incidence of developing cervical dysplasia and cancer, immune dysfunction from HIV infection has a greater causal impact on infection with HPV and low-grade cervical dysplasia compared to development of higher-grade dysplasia, consequently leading to a higher prevalence of lower-grade cervical lesions in women with HIV [19]. Therefore, using a screen-and-treat strategy in this population can lead to overtreatment of cervical dysplasia [18].

Besides HPV testing, visual inspection with acetic acid is a widely used screening method in several lower resource settings in Africa, South America, and Asia. It is a preferred option for screening in many low to middle income countries due to its accessibility, low price, and instant delivery of results [20]. However, the test has varying sensitivity and efficacy that can depend on the administering provider [21, 22]. In a large randomized control trial of about 70,000 women in Mumbai, India, visual inspection with acetic acid screening over a 12-year period decreased overall mortality from cervical cancer, but did not significantly decrease overall incidence of cervical cancer [23].

While HPV DNA tests are currently too expensive to be a feasible option in most low resource settings [24], implementation of an HPV-based screening program in low to middle income countries would lead to an accelerated decline of cervical cancer incidence and deaths and be more cost-effective in the long run [22, 25]. In a modelling analysis of 78 low to middle income countries by Simms et al., HPV screening every five years was the most efficacious and cost-effective method of screening [22], while visual inspection with acetic acid or cytology screening were both less efficacious and less cost-effective [22]. Furthermore, in 2022 the International Agency on Research on Cancer reconvened with 27 scientists from 20 countries to update the Handbook of Cervical Screening. The Agency concluded that, weighing all benefits and harms, primary HPV testing was the most effective screening tool for cervical cancer [25]. Still, as mentioned above, the immediate test cost remains a barrier to implementing HPV testing globally.

Considering that, novel screening strategies and protocols that are low-cost and can be implemented in a point-of-care setting are still needed. These strategies must address multiple barriers to implementation. The narrative review below addresses such barriers, techniques, and technologies that have been implemented in low to middle income countries thus far.

## Lack of Awareness and Knowledge About Cervical Cancer

There are several barriers to implementing a successful cervical cancer screening program in low to middle income countries. At an individual level, there is substantial lack of awareness and knowledge about cervical cancer and screening for cervical cancer in lower resource settings [26, 27]. Many women have a fatalistic view of cervical cancer, believing a positive screening test to mean incurable terminal illness [27, 28]. In a systematic review of barriers to cervical cancer screening in low to middle income countries, the lack of awareness of cervical cancer was found to be the most important barrier to screening across regions [29].

To increase awareness of cervical cancer programs in lower resource settings, more education programs can be implemented. According to a systematic assessment of cervical cancer educational intervention programs across the world, several different interventions are effective in increasing cervical cancer awareness [30]. This includes remote health education techniques like calls and social media posts [30]. Another systematic review and meta-analysis examining the effects of cervical cancer education on screening rates found that theory-based educational interventions increased screening rates by almost double by addressing health beliefs and misconceptions [31].

## Fears About Pelvic Examination and Sexual Health Stigma

At a societal level, there can be embarrassment associated with getting screened for a disease associated with sexual or reproductive health. In multiple lower income countries, including China, India, and Nigeria, women expressed the need for permission from their spouse or families to be screened, irrespective of religion (30). In one systematic review, 33% of studies in Africa, Asia, and South America reported women were embarrassed to either get screened or undergo a pelvic examination [27].

Novel collection techniques that have been implemented in low to middle income countries can increase privacy and eliminate the need for a pelvic examination.

Numerous countries have introduced or piloted vaginal self-sampling for HPV-based tests [32, 33]. With a self-swab, a woman can test herself at her own convenience in a private environment. She also does not have to undergo an invasive procedure or interact with health personnel. Several feasibility studies have found HPV self-sampling to be acceptable and preferable for women in in Asia, Africa, and Latin America [34, 35]. For example, one meta-analysis of HPV self-sampling acceptability studies in Latin America showed an acceptability of up to 80% [36]. Self-sampling is also more cost-effective, as it reduces the need for trained personnel and office examinations [32].

After a self-sample is collected, the sample can either be processed in a separate laboratory or a point-of-care setting for further triage or treatment. In 2022, Papua New Guinea was the first low to middle income country to evaluate the clinical validity of HPV self-sampling combined with same-day provider-administered thermal ablation [37]. It was found that this intervention was highly acceptable among women and accurately detected and treated HPV-related cervical lesions [37].

Urine testing also provides a less invasive alternative to cervical or vaginal swabs for detection of HPV in women. However, unlike vaginal self-sampling, urine testing has not yet been implemented in any low to middle income country. In general, there is comparable sensitivity and specificity between urine self-sampling and clinician-collected cervical swabs for detecting cervical intraepithelial neoplasia 2+, with a sensitivity of 88% and a specificity of 77% for urine testing in a recent meta-analysis [38]. Barriers to implementing urine testing include lack of both commercial standardization for urine-based HPV tests and regulatory approval for an HPV assay for urine samples [39]. Moreover, high-output molecular analyzers used for HPV sample processing do not yet incorporate urine samples [39]. Research, however, in this area is progressing, and certain manufacturers are in the process of bringing urine-based HPV tests to the market.

## Paucity of Health Care Services and Trained Providers

Overall in lower resource settings, there is a supply-demand gap for health care and screening services [24]. Women travel long distances to reach a clinic with long wait times and limited follow up, especially in rural areas [40, 41]. In some regions, only specialized centers offer cervical cancer screening [42]. Furthermore, health care workers in these clinics are often not knowledgeable or trained sufficiently, and appropriate equipment may not be available [41]. Cervical cancer screening requires multiple levels of training, depending on the type of screening protocol being utilized.

For instance, for a pap smear, an initial pelvic examination requires a trained provider. The sample must then be analyzed by a trained cytopathologist. Colposcopy with visual inspection does not require a pathologist, but a trained health care worker must be able to interpret the results appropriately. For HPV testing, a central laboratory usually must process samples. If significant cervical dysplasia is found for either method, a trained medical provider must treat the lesion.

## Lack of Clear Policy and Screening Guidelines

Among both patients and healthcare workers, the lack of clear policy and guidelines present ongoing barriers to effective population-based screening at scale [27]. As mentioned above, only 41% of low to middle income countries have an actual funded cervical cancer screening strategy in operation, and screening is often opportunistic, meaning there is no national organized n screening invitation protocol for the target population [5, 6]. Cytology-based screening strategies have been largely unsuccessful in low to middle income countries due to lack of operational capacity and health care infrastructure [43]. Several low to middle income countries have piloted screening strategies that address cost and infrastructure barriers.

One cost-effective option is a visual inspection-based screening program. Both Bangladesh and Malawi, for instance, have implemented visual inspection with acetic acid-based screening programs due to its affordability [44]. Bangladesh adopted a screen-triage-treat approach in 2004; visual inspection-positive cases are referred for colposcopy for further treatment [45]. However, screening coverage remains low, at about 11% [45]. On the other hand, Malawi, which has one of the highest cervical cancer mortality rates worldwide of 51.5 deaths per 100,000 per year, deployed a screen and treat strategy in 2015 [46]. Visual inspection-positive women are treated with cryotherapy or thermocoagulation [46]. Still, according to the Malawi Ministry of Health in 2021, only 34% of eligible women had been screened in the previous year [46]. Low uptake in Malawi is largely explained by “supply-side” barriers related to difficulties accessing the health care system [46].

Still, WHO recommends cervical HPV-based testing as the preferred screening method. China recently implemented visual inspection with acetic acid as a triage test for HPV-positive cases, the first screening protocol of its kind [47]. This protocol was implemented between 2016 and 2021 in Ordos City, an ethnically diverse and resource-limited region with low cervical cancer screening rates [47]. The program was implemented on a large scale, covering about 187,863 women [47]. By using a point-of-care HPV test and subsequently performing visual inspection triage, this strategy immediately evaluated women and linked them to care. Among targeted women, screening coverage rate was 92.4% [47]. However, the cost-effectiveness of this strategy has not yet been studied.

Conversely, Indaiatuba, a city in Brazil with a population of 240,000, replaced their opportunistic cytology screening program with an organized primary HPV testing with colposcopy triage in 2017 [48]. This strategy collected specimens for HPV testing at multiple primary care sites throughout the city [48]. These tests were all sent to one central laboratory in the city [48]. Using one central laboratory streamlined processing of the HPV test and allowed all screening information to be present in one database. After five years of implementation, the screening coverage rate increased to 58.7% among 35,000 women between ages 25–64, including the pandemic years where screening coverage decreased [49].

## Solutions and Examples of Novel Cervical Cancer Screening Strategies and Devices

Several lower resource settings do not have the infrastructure to support a high level of triage and training, and those barriers decrease the incentive for women to get screened and treated. However, some novel methods can help reduce those barriers by providing more direct delivery of health care services to patients and eliminate the need for trained providers to increase screening uptake.

As described above, HPV self-sampling minimizes interaction with the health care system by eliminating the need for a pelvic examination. Generally, self-sampling kits are distributed by an “opt-in” or “opt-out” strategy [32]. In the opt-in strategy, patients are invited to undergo specimen self-collection via mail invitation [32]. In the opt-out strategy, patients are directly mailed a self-sampling kit [32]. While the opt-out strategy is more expensive, it translates to higher screening uptake [32]. However, the opt-in strategy can be optimized with effective ordering platforms [32].

New devices and technologies have been developed that can reduce the burden on a health care system when used in conjunction with existing screening protocols. With the ubiquity of smart phones globally, some studies have employed smart phones to transmit local visual inspection images taken by a tertiary healthcare worker to a remote expert [50]. This strategy was found to be effective in small-scale studies in Tanzania, Madagascar, and Ghana [51–53].

The callascope was developed by scientists at the Duke University with the goal of creating a new screening device that would accurately image the cervix without the need for a pelvic exam [40, 54]. It is a light, small device shaped like a tampon that can be inserted into a woman’s vagina to take images of the cervix [55]. This device can be self-inserted or inserted by a community health worker, which minimizes discomfort associated with speculum insertion and reduces the burden associated with training of medical providers [40, 54]. Since its conception, the callascope has been updated to produce more accurate images [40, 54]. Clinical studies assessing the feasibility of the callascope have been conducted in the U.S., Ghana, Peru, and Tanzania [40, 54]. The callascope is also much cheaper than the colposcope; the manufacturing cost of callascope is about 500 U.S. dollar, whereas the cost of manufacturing a new colposcope is 20,000 U.S. dollars [56]. The callascope device has been shown to have comparable imaging quality to a more expensive colposcope [40].

High-resolution microendoscopy is an in-vivo method to assess cervical dysplasia that has lower-costs than traditional colposcopy and could be implemented in low-resource settings [57]. To use HRME, first a solution of proflavin, a fluorescent DNA label, is applied to the cervix. Then, using a speculum, a probe is inserted into the cervix to capture an image. High-resolution microendoscopy allows for highly accurate real-time analysis of cervical specimens without the necessity of staining, with comparable sensitivities and specificities to colposcopy [57]. Larger studies to assess feasibility are currently being undertaken in South America, Latin America, and the U.S [21]. One prospective trial in rural Brazil delivered high-resolution microendoscopy via a mobile van. Sensitivity and specificity were comparable to the colposcope, and real-time diagnostic evaluation was provided [58]. A major drawback of high-resolution microendoscopy is that it still requires speculum insertion.

There is also a growing interest in utilizing artificial intelligence and machine learning algorithms to expedite analysis of biopsies. An algorithm could learn certain aspects of cervical dysplastic lesions to assist with diagnosis and could address the deficit of trained pathologists needed to analyze biopsies in lower resource settings [59]. Artificial intelligence-assisted colposcopy can decrease instances of misdiagnoses and improve accuracy of biopsy analysis [59, 60]. To make artificial intelligence a feasible option for cervical cancer screening in the future, more clinical data is needed to increase the robustness of machine learning algorithms [60]. This can help improve the diagnostic differentiation and accuracy of artificial intelligence in a way that can replace the diagnostic capabilities of a physician. Of note, no large-scale implementation studies have been conducted with artificial intelligence technology in low to middle income countries yet.

## HIV and Cervical Cancer

Women with HIV have a six times higher risk of developing cervical cancer than women without HIV [61, 62]. HIV-related immunosuppression makes it more difficult to clear HPV infection, resulting in a greater frequency of persistent infection, which increases the risk of developing cervical cancer [61]. Of women with HIV and cervical cancer, 85% live in sub-Saharan Africa [61]. Given the prevalence of this subpopulation in certain low to middle income countries, different cervical cancer screening strategies are employed. The WHO recommends more frequent cervical cancer screening intervals for women living with HIV, advising screening every 3 years rather than every 5 to 10 years, and also advised that women with HIV start screening at 25 rather than 30, the age recommended for the general population [3, 17]. Expanding cervical cancer screening access for women living with HIV is essential for cervical cancer prevention in low to middle income countries. Particularly, integration of cervical cancer screening into existing HIV services, or vice versa, has been shown to be effective and acceptable in low to middle income countries [63]. Integration is supported by major worldwide funders like The United States President’s Emergency Plan for AIDS Relief and The Global Fund [64, 65] However, there is sparse data on the impact of integrated programs on long-term outcomes for cervical cancer for women with HIV [63]. Further data regarding efficacy of new cervical cancer screening technologies and strategies like screen-and-treat are needed to elucidate the most effective prevention methods for cervical cancer for women living with HIV.

## Conclusion

This review discusses emerging strategies and technologies that have been implemented in low to middle income countries to address barriers to cervical cancer screening. The ideal test for implementation in low to middle income countries would be a low-cost, point-of-care rapid HPV test.

In addition, it is essential to emphasize the importance of primary care and community health in developing a sustainable screening strategy for the future. Investment into community-based programs and health care workers could increase local involvement and cost-effectiveness.

While accessible secondary screening methods reduce cervical cancer cases and mortality, the HPV vaccine is still the best method available for primary cervical cancer prevention. One model suggests that if the HPV vaccine is given to over 90% of the eligible population in combination with high screening coverage, cervical cancer could be eliminated by the end of the century [66]. While WHO recommends the HPV vaccine for all girls aged 9 to 14 years old, less than 33% of low to middle income countries have presented a plan for HPV vaccination [67, 68].

However, the cost of vaccination has steadily decreased over the years, increasing optimism. Moreover, Gavi, the Vaccine Alliance, WHO, and UNICEF announced in 2023 that they are funding HPV vaccination programs in low to middle income countries for as little as $4.50 USD per vaccine dose [69].

Therefore, a complete cervical screening strategy in low to middle income countries is a combined armament of routine HPV vaccination, a low-cost point-of-care HPV test, and low-cost screening and imaging. Continued investment into organized screening strategies can allow settings with high cervical cancer rates to have long-lasting and effective screening programs for the future.

## Key References

1. WHO guideline for screening and treatment of cervical pre-cancer lesions for cervical cancer prevention 2nd ed. Geneva 2021.
  These guidelines from the World Health Organization endorsed the screen-and-treat approach for cervical cancer screening to address resource disparities in low to middle income countries. These guidelines also include screening recommendations for women living with HIV.
2. Bouvard V, Wentzensen N, Mackie A, Berkhof J, Brotherton J, Giorgi-Rossi P, et al. The' Perspective on Cervical Cancer Screening. N Engl J Med. 2021;385(20):1908-18.
  This special report from the International Agency for Research on Cancer determined that HPV DNA testing was the most effective form of cervical cancer screening.
3. Petersen Z, Jaca A, Ginindza TG, Maseko G, Takatshana S, Ndlovu P, et al. Barriers to uptake of cervical cancer screening services in low-and-middle-income countries: a systematic review. BMC Women’s Health. 2022;22(1).
  This systematic review highlights multiple barriers to implementation of cervical cancer screening services in low to middle income countries.
4. Sigfrid L, Murphy G, Haldane V, Chuah FLH, Ong SE, Cervero-Liceras F, et al. Integrating cervical cancer with HIV healthcare services: A systematic review. PLOS ONE. 2017;12(7):e0181156.
  This systematic review provides evidence for the benefit of integrating cervical cancer services with existing HIV healthcare services in low to middle income countries.

## Data Availability

No datasets were generated or analysed during the current study.
